# Immobilized enzymes in flow-injection analysis: present and trends

**DOI:** 10.1155/S1463924688000069

**Published:** 1988

**Authors:** J. Ruz, F. Lázaro, M. D. Luque de Castro

**Affiliations:** Department of Analytical Chemistry Faculty of Sciences University of Córdoba Córdoba Spain

## Abstract

An overview of the use of immobilized enzymes in flow-injection analysis (FIA) is presented. The joint use of FIA and immobilized enzymes means that analytical procedures are easily automated, analytical costs are reduced and methods are faster. The future possibilities for this combination are discussed.


Journal of Automatic Chemistry, Vol. 10, No. 1 (January–March 1988), pp. 15–19

### Immobilized enzymes in flow-injection analysis: present and trends

**J. Ruz, F. Lázaro and M. D. Luque de Castro**

*Department of Analytical Chemistry, Faculty of Sciences, University of Córdoba, Córdoba, Spain*

*An overview of the use of immobilized enzymes in flow-injection analysis (FIA) is presented. The joint use of FIA and immobilized enzymes means that analytical procedures are easily automated, analytical costs are reduced and methods are faster. The future possibilities for this combination are discussed.*

### Introduction

There are two reasons for limiting the use of enzymes in analysis: instability and price; these can be overcome by placing them onto water-insoluble matrices during the analytical process (i.e. by immobilization).

The enzyme immobilization technique was first developed in 1916 [1], all further development was during the last decade [2 and 3]. Immobilization methods can be classified according to the strength of the matrix-enzyme linkage into physical methods (involving no bond) and chemical methods, where a covalent bond is formed either between the enzyme and the support, or between pairs of protein molecules. Although physical methods are less complex and laborious, the linkage involved is labile and the system loses activity over time. The formation of a covalent bond in chemical methods, however, provides the enzyme with nearly natural environment; so these are the most commonly used immobilization procedures.

Enzymatic analysis has been widely used in automatic flow methods [4–6], particularly in Flow Injection Analysis (FIA) [7–10]. The variety of FIA methods using immobilized enzymes proposed in the last few years shows how useful they are in many areas of application. The benefits offered by the joint use of FIA and immobilized enzymes will be applicable to a number of, to date undiscussed, areas and these are described in this article.

### Fundamentals of enzyme immobilization in flow-injection analysis

Of the two chief types of enzyme immobilization procedures, physical linkage by adsorption has rarely been used in FIA because its low stability means that it is unsuitable for flow systems. The linkage, however, can be strengthened by using a mediator such as N,N-dimethyl-7-amino-1,2-benzophenoxazinium ion [11]. This is adsorbed onto a carbon electrode, which thus becomes more receptive to the enzyme. Neither membrane nor gel trapping has been used in FIA, although Olsson *et al.* have developed an electrode accommodating an enzyme trapped between two cellulose dialysis membranes [12]. Immobilization by covalent bonding has been the most common technique used in FIA.

Both inorganic and natural and artificial organic supports have been used: glass, cellulose and nylon, respectively, being the most popular. Of the methods proposed to date, 64% have used an inorganic support; 29% have used organic supports and the remainder used a combination [13–15]. The most popular organic matrix is glass, which is used as beads of various sizes [14 and 16–21], or as controlled-pore glass (CPG) [22–32]. Platinum has been used much less frequently, with the twofold function of support and working electrode for amperometric determinations [13, 15 and 33–35], and so has carbon [11, 36, 37]. Only once has $IrO_2$ activated with cyanuric chloride been used as support [38]. Among organic supports, the commonest is nylon [14, 15 and 39–43], followed by a series of polymers and gels of different characteristics, such as propylene [44], sepharose [44–48], silica gel [13 and 49–52], octylagarose gel [46], wakogel C-100 [50], polyamide gel [53], aminocellulofine [54], glycophase [30] etc. Figure 1 shows the variation in the use of the most popular supports over the period 1978–1986.

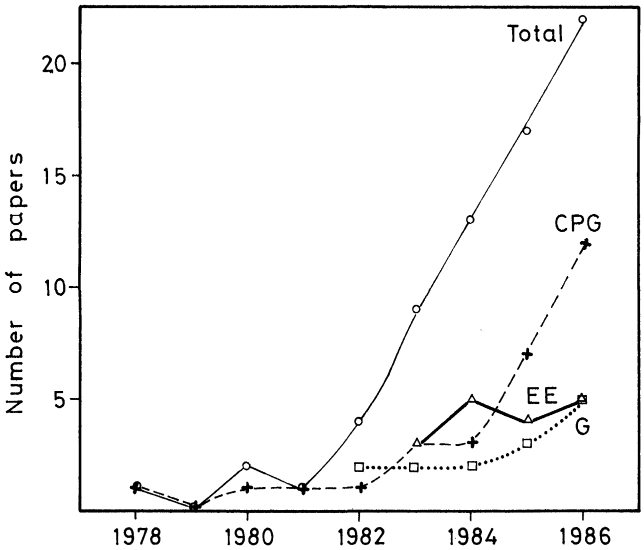

*Figure 1. Variation of the annual number of FIA methods using immobilized enzymes: Overall (○) and methods using controlled pore glass (CPG, +), enzymatic electrodes (EE, △) or gels (G, □).*

Of the three basic steps involved in the immobilization process (activation or functionalization, coupling of the binding agent and enzyme immobilization) the first is the most flexible. Inorganic supports are usually functionalized with aminosilane, $X_3$-Si-$NH_2$, which bears a terminal amino group for coupling with glutaraldehyde in the following step [13, 16–28, 31–35, 37, 51, 52 and 55–78]. 2,2,2-trifluoroethane sulfonyl chloride [79], carbodiimide [36], dichloro dimethyl silane+ethylendiamine [14], p-benzoquinone [29], cyanuric chloride [38] and sodium periodate (later reduced with $NaBH_4$) [30] have also been used for this purpose. The last agent has been used with some organic supports such as silica gel [49–52].

The functionalizer most frequently used with organic supports such as nylon is dimethyl sulfate [15, 39, 40, 42 and 43]; although triethylozonium tetrafluoroborate (to which lysine or 1,6-diaminohexane is subsequently added) [14] has also been used. The rest of the supports –generally gels– are functionalized with cyanogen bromide [44, 46 and 47].

Whatever the functionalizer used, the second step always involves the coupling with glutaraldehyde. In most cases (70% of all papers reported on the subject) the enzyme-support set is packed in a tube making the immobilized enzyme reactor (IMER), where interstitial dead volumes are kept to a minimum so that the key role in the development of the analytical reaction is played by the flow-rate rather than by the time. The enzymatic reaction occurring in the reactor is generally fast; this allows the use of very short lengths, which minimize over-pressure resulting from packing in the FIA system. An alternative approach is the open tubular heterogeneous enzyme reactor (OTHER), which is prepared by immobilizing the enzyme onto the inner surface of a nylon tube [14, 41 and 43] or onto other material [16, 18 and 70). A third type of reactor, developed by Coulet, is the so-called 'membrane reactor' which is made of nylon and on which the enzyme is immobilized by covalent bonding. This type of reactor is usually supported on an electrode [15, 39, 40, 42 and 44]. Occasionally, the enzyme is used unbound, namely immobilized by being physically trapped between two membranes (sandwich-type) [12]. Finally, the variable location of the immobilized enzyme on the electrode endows FIA methods with great versatility. The electrode is usually Pt [13, 15, 33, 34 and 44], carbon [11, 36 and 37] or $IrO_2$ [38].

The most serious shortcomings in the use of immobilized enzymes in FIA arise from the nature of the immobilization processes itself: the long time gap between the steps involved and the differences between the optimum pH zones for enzyme stability and reaction development.

### Applications of immobilized enzymes to flow-injection analysis

Although immobilized enzymes have been used in chemical analysis for over a decade [3], their joint application with flow-injection methods dates from only 1982. The selectivity and low analytical cost of immobilized enzymes are perfect complements to the modular character, low sample consumption and speed of the FIA technique, which thus offers a greater potential than with the former use of dissolved enzymes [80].

Although FIA methods involving immobilized enzymes (IEs) have been applied to a variety of analytes, over 30% of the papers published on the topic (27 papers) deal with the determination of glucose. This is basically the result of the characteristics of glucose oxidase (low cost, good stability of the linkage to the support, etc.), which makes it suitable for testing the behaviour of a new configuration, reactor or detection system, and of glucose being one of the parameters most frequently determined in clinical laboratories, as well as in food analysis. The most frequent technique for detection of this analyte has been amperometry (17 methods), with enzymatic [39] or non-enzymatic [67] electrodes. Enthalpimetry [69], fluorimetry [59], chemiluminescence [65] and photometry [41], have been used less frequently both in the clinical [29] and nutritional [42] areas (in the latter case in individual or simultaneous analysis with sucrose [62] or fructose [59]). Owing to the complexity of the sample matrices encountered, the presence of such interferents as ascorbic or uric acid calls for the separation of the analyte by different procedures [35, 49 and 66]. Other carbohydrates such as sucrose [31, 62, 67 and 76], galactose [32, 49 and 63], lactose [37 and 63], fructose [54 and 59] or starch [71] have also been determined by FIA methods involving IEs, particularly in the nutritional area and with amperometric detection. Other analytes typically determined are of special interest to the clinical field (cholesterol [14, 46, 49, 51, 56, 65 and 74], lactic acid [16, 40, 50, 52, 65 and 81], uric acid [23, 24, 68 and 82], creatinine [43, 73 and 78] and urea [27, 28 and 75] or forensic chemistry (ethanol [48, 55, 57, 58, 64 and 81]). Several methods have been proposed for the determination of the activity of different dissolved [33, 48 and 77] or immobilized [38] enzymes, as well as for the direct determination of co-enzymes in biological fluids ($NAD^+$ [17], ATP [77], NADPH and $FMNH_2$ [47]. Little attention has so far been paid to the analysis for vitamins [45] and inorganic species such as Zn(II) [26], sulphite [72], pyrophosphate [48] or hydrogen peroxide [21, 25 and 79].

The frequency of use of the various kinds of detection techniques in methods involving IEs-FIA is shown in figure 2. Both electroanalytical and optical techniques have been used. The somewhat commoner use of amperometry is probably the result of the good redox properties of enzymatic reaction products: detected either directly [66] or through a coupled reaction [39]. Potentiometry has been applied only with pH electrodes [20], or ammonia-selective electrodes [73]. Although photometry has been the most popular of optical technique, fluorimetry is gaining acceptance because it has higher sensitivity and selectivity. Other detection techniques less frequently used, but with a great potential, are chemiluminescence and bioluminescence, particularly when the enzyme is immobilized in the flow-cell [78].

Flow-injection methods using IEs are most frequently applied to clinical analysis (45% of all methods proposed to date), although they are being increasingly used in food analysis (15%). Both areas have benefitted from the high selectivity of enzymatic reactions as applied to complex matrices such as blood (whole [58], serum [65] and plasma [61]), urine [75] and to all types of foods and

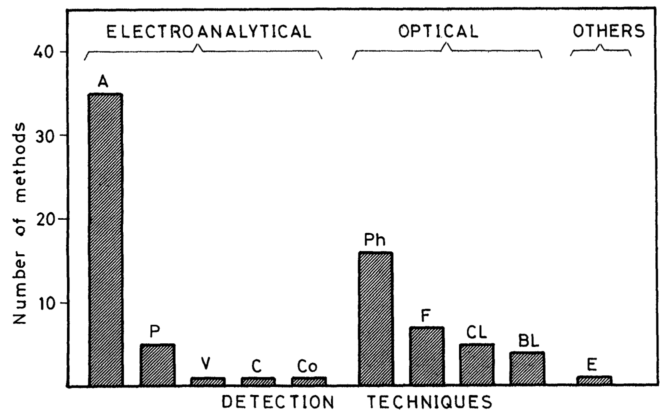

*Figure 2. Variation of the number of FIA methods using IEs and the different detection techniques. *A = amperometry; P = potentiometry; V = voltammetry; C = coulometry; Co = conductimetry; Ph = photometry; F = fluorimetry; CL = chemiluminescence; BL = bioluminescence; E = enthalpimetry.*

drinks (for example, wine [55], milk [63], soft drinks [67], butter [75]). It is surprising to discover that these methods are rarely used in some areas, such as industrial process control [60 and 74] and pharmaceutical analysis [20], where the stability of immobilized enzymes, added to their low cost and easy handling, should make them particularly appealing.

FIA methods are not often used for multideterminations with IEs, despite the frequent need to control more than one parameter in the different areas of application and the simplicity of using parallel reactors with different immobilized enzymes. This has been discussed for food analysis for determining glucose–sucrose in soft drinks [31, 62, 67], L- and D-lactic acids [52], ethanol-acetaldehyde in wine [55] and glucose–fructose in fruit juices, yoghourt and dessert powder [59], but only one simultaneous determination (free and sterified cholesterol in blood) has been proposed in the clinical area [51]. These determinations can be carried out by sequential [59, 62, 67 and 76] or simultaneous [55] injection of two sample plugs undergoing different reactions, or by injection of a single sample plug, which is split into two subplugs prior to arrival at the enzymatic reactors [52, 67 and 76].

### Ways of increasing selectivity in IEs-FIA

At least 40% of the methods proposed to date have no practical application. Despite the high selectivity of enzymatic reactions (further increased by immobilization), the matrix complexity often demands ever higher selectivity. This can be done in several ways:

(1) By separation of the analyte by on-line coupling of a suitable unit (dialyser [27, 32, 41, 63 and 66] or liquid chromatograph [26]).

(2) By using a reaction coupled with the enzymatic one to increase the monitoring wavelength in photometric [32] or fluorimetric [61] methods, or by decreasing the applied potential in amperometric detection [39].

(3) By using a suitable reactor to eliminate interferents. It can be an enzymatic reactor removing species present in the sample and interfering with the monitored reaction product for example $NH_3$ in the determination of creatinine [43 and 73], or degrading a species with redox properties similar to those of the analytes (for example ascorbic acid in the determination of fructose [54]). The reactor can also consist of a silica gel column modified to immobilize the Cu(II)-diethyldithiocarbamate complex [33, 35, 63 and 66] or Cu(II) [18] with the aim of eliminating reductants. This is also the function of the electrolytic column proposed by Yao *et al.* [49 and 83].

(4) By using differential measurements, i.e. by taking the difference between the signal provided by the sample in the presence and absence of analyte [45] or of the enzymatic reaction [60 and 82], achieved by sequential injection of two aliquots of sample [45 and 60], by splitting the injected sample [82] or by simultaneous injection of two sample plugs as the analytical signal. The use of two injection valves [84 and 85] coupled with the enzymatic reactor in the loop of the secondary valve is a recent alternative to these differential measurements [86]. A variant of these differential measurements involves monitoring the conductivity of the sample plug before and after the enzymatic reactor [75].

(5) A new, unpublished alternative is the kinetic monitoring in the stopped-flow/FIA mode, i.e. the monitoring of the enzymatic reaction when the reactant plug is stopped in the flow-cell. In this way, amperometric monitoring can be performed with chemically modified electrodes accommodating an immobilized enzyme. Alternatively, the development of absorbance or fluorescence can be monitored with the aid of an enzyme immobilized on CPG or gel and introduced into the flow-cell, or with the enzyme fixed on the walls of a suitable flow-cell. All these variants are currently being investigated.

Thus, although the commonest location of the immobilized enzyme is in the reactor (between the injection and detection system), figure 3, it can also be placed in the injection valve loop or in the flow-cell.

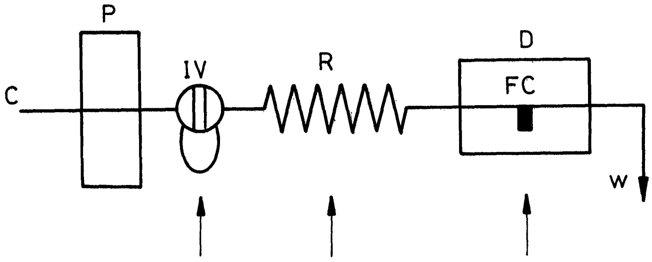

*Figure 3. Possible locations of the immobilized enzyme in an FIA system. C = carrier, P = peristaltic pump; IV = injection valve; R = reactor; FC = flow-cell; W = waste.*

### Trends

The use of enzymes immobilized on CPG or gel contained in the flow-cell for chemi- [19] or bio-luminescent [47, 48 and 77] monitoring is becoming more and more frequent. This is the optimum location for this type of reaction in which the transient characteristics of the monitored product must be developed by the time it passes through the detector. Nevertheless, it could be interesting to use this location in conjunction with the kinetic stopped-flow mode and other detection techniques (photometry, fluorimetry) allowing the reaction product to be monitored for longer times. The enzyme can also be immobilized on the flow-cell walls. The use of enzymatic electrodes for kinetic measurements can be very useful to overcome interferences.

The advantages of immobilized enzymes (higher stability, longer life span, lower analytical cost and higher selectivity) over dissolved enzymes make them suitable for routine analysis (clinical, food and pharmaceutical analysis), especially by exploiting the possibility of performing simultaneous determinations, and for industrial process control in conjunction with FIA, which also contributes rapidity, precision, financial cost savings and easy automation.

Another way of complementing the IEs-FIA coupling is the immobilization of non-enzymatic reagents in the flow-cell and on the above-mentioned supports. As for enzymes, for immobilizing reagents, only CPG (luminol [87] and aminofluoranthene [88]) associated with chemiluminescence detection have been used as support. Nevertheless, its use associated with other optical techniques can be of substantial benefit. Another promising development is the monitoring of the absorbance, reflectance or fluorescence of an ion-exchanger [89] confined in the flow-cell, on which a coloured or fluorescent product is transiently retained, or the analyte with which the reagent is later reacted, or on which the reagent that reacts with the analyte is permanently immobilized.

As can be seen, there is a wide range of unexplored possibilities in this area, towards which is oriented the future of immobilized enzymes in FIA.

### References

1. Nelson, J. M. and Griffins, G. J., *American Chemical Society*, **38** (1916), 1109.
2. Carr, P. W. and Bowers, L. D., *Immobilized Enzymes in Analytical and Clinical Chemistry* (Wiley Interscience, New York, 1980).
3. Guilbault, G. G., *Analytical Uses of Immobilized Enzymes* (Marcel Dekker, New York, 1984).
4. Kunz, H. J. and Stasny, M., *Clinical Chemistry*, **20** (1974), 1018.
5. Dahodwala, S. K., Wibel, M. K. and Humphrey, A. E., *Biotechnology and Bioengineering*, **18** (1976), 1679.
6. Voleski, B., and Edmond, C., *Biotechnology and Bioengineering*, **21** (1979), 1251.
7. Rocks, B., and Riley, C., *Clinical Chemistry*, **28** (1982), 409.
8. Riley, C., Rocks, B., and Sherwood, R. A., *Talanta*, **31** (1984), 879.
9. Linares, P., Luque de Castro, M. D. and Valcárcel, M., *Reviews in Analytical Chemistry*, **VIII** (1985), 229.
10. Yao, T., *Journal of Flow Injection Analysis*, **22** (1985), 115.
11. Marko-Varga, G., Appelquist, R. and Gorton, L., *Analytica Chimica Acta*, **179** (1986), 371.
12. Olsson, B., Lundback, H., Johansson, G., Scheller, F. and Nentwing, J., *Analytical Chemistry*, **58** (1986), 1046.
13. Yao, T., Sato, M., Kobayashi, Y. and Wasa, T., *Analytica Chimica Acta*, **165** (1984), 291.
14. Yoshida, S., Cho, T. and Hirose, S., *Bunseki Kagaku*, **31**, 2 (1982), 77.
15. Moody, G. J., Sanghera, G. S. and Thomas, J. D. R., *Analytical Proceedings*, **23** (1986), 446.
16. Morishita, F., Hara, Y. and Kojima, T., *Bunseki Kagaku*, **33**, 12 (1984), 642.
17. Chow, T., Yoshida, S., Itoh, M., Hirose, S. and Takeda, T., *Bunseki Kagaku*, **33**, 6 (1984), 310.
18. Kojima, T., Hara, Y. and Morishita, F., *Bunseki Kagaku*, **32**, 4 (1983), E101.
19. Hara, T., Toriyama, M. and Imaki, M., *Bulletin of the Chemical Society of Japan*, **55**, 6 (1982), 1854.
20. Ghanasekaran, R. and Mottola, H. A., *Analytical Chemistry*, **57** (1985), 1005.
21. Hwang, H. and Dasgupta, P. K., *Mikrochim. Acta*, III (1985), 77.
22. Appelquist, R., Marko-Varga, G., Gorton, L., Torstensson, A. and Johansson, G., *Analytica Chimica Acta*, **169** (1985), 237.
23. Yao, T., Sato, M. and Wasa, T., *Nippon Kagaku Kaishi*, February, **2** (1985), 189.
24. Tubata, M., Fukunaga, C., Ohyaby, M. and Murachi, T., *Journal of Applied Biochemistry*, **6**, 4 (1984), 251.
25. Olsson, B. and Ogren, L., *Analytica Chimica Acta*, **145** (1983), 87.
26. Risinger, L., Ogren, L. and Johansson, G., *Analytica Chimica Acta*, **154** (1983), 251.
27. Gorton, L. and Ogren, L., *Analytica Chimica Acta*, **130** (1981), 45.
28. Adams, R. E. and Carr, P. W., *Analytical Chemistry*, **50** (1978), 944.
29. Lomen, C. E., Uditha de Alwis, U. and Wilson, G. S., *Journal of the Chemical Society Faraday Transactions*, **82**, 4 (1986), 1265.
30. Toyoda, T., Kuan, S. S. and Guilbault, G. G., *Analytical Chemistry*, **57** (1985), 2346.
31. Masoom, M. and Townshend, A., *Analytical Proceedings*, **22** (1985), 6.
32. Olsson, B., Lundback, H. and Johansson, G., *Analytica Chimica Acta*, **167** (1986), 123.
33. Yao, T., *Analytica Chimica Acta*, **153** (1983), 169.
34. Yao, T., Nakarishi, K. and Wasa, T., *Bunseki Kagaku*, **33**, 4 (1984), 213.
35. Yao, T., *Analytica Chimica Acta*, **153** (1983), 175.
36. Wieck, H. J., Heider, G. M. and Yacynych, A. M., *Analytica Chimica Acta*, **158** (1984), 137.
37. Matsumoto, K., Hamada, O., Ukeda, H. and Osajima, Y., *Agricultural Biology and Chemistry*, **49**, 7 (1985), 2131.
38. Osborn, J. A., Yacynich, A. M. and Roberts, D. C., *Analytica Chimica Acta*, **183** (1986), 287.
39. Moody, G. J., Sanghera, G. S. and Thomas, J. D. R., *Analyst*, **112** (1987), 65.
40. Mascini, M., Moscone, D. and Palleschi, G., *Analytica Chimica Acta*, **157** (1984), 45.
41. Worsfold, P. J., Farelly, J. and Matharu, M. S., *Analytica Chimica Acta*, **164** (1984), 103.
42. Moody, G. J., Sanghera, G. S. and Thomas, J. D. R., *Analyst*, **111** (1986), 605.
43. Mascini, M., Fortunati, S., Moscone, D. and Palleschi, G., *Analytica Chimica Acta*, **171** (1985), 175.
44. Pacakova, V., Stulik, K. and Brabcova, D., *Analytica. Chimica Acta*, **159** (1984), 71.
45. Bradberry, C. W. and Adams, R. N., *Analytical Chemistry*, **55** (1983), 2439.
46. Karube, I., Hara, K., Matsuoca, H. and Suzuki, S., *Analytica Chimica Acta*, **139** (1982), 127.
47. Nabi, A. and Worsfold, P. J., *Analyst*, **111** (1986), 1321.
48. Ibid., *Analytical Proceedings*, **23** (1986), 415.
49. Yao, T. and Kobayashi, Y., *Bunseki Kagaku*, **32**, 4 (1983), 253.

50. Yao, T., Kobayashi, Y. and Musha, S., *Analytica Chimica Acta*, **138** (1982), 81.
51. Yao, T., Sato, M., Kobayashi, Y. and Wasa, T., *Analytical Biochemistry*, **149** (1985), 387.
52. Yao, T. and Wasa, T., *Analytica Chimica Acta*, **175** (1985), 301.
53. Uchiyama, S., Tohfuku, Y. Suzuki, S. and Muto, G., *Bunseki Kagaku*, **35**, 2 (1986), 134.
54. Matsumoto, K., Homada, O., Uqueda, H. and osajima, Y., *Analytical Chemistry*, **58** (1986), 2732.
55. Lázaro, F., Luque de Castro, M. D. and Valcárcel, M., *Analytical Chemistry*, **59** (1987), 1859.
56. Fernández-Romero, J. M., Luque de Castro, M. D. and Valcárcel, M., *Clinica Chimica Acta*, **167** (1987), 97.
57. Ruz, J., Luque de Castro, M. D. and Valcárcel, M., *Analyst*, **112** (1987), 259.
58. Ruz, J., Luque de Castro, M. D. and Valcárcel, M., *Talanta* (in press).
59. Linares, P., Luque de Castro, M. D. and Valcárcel, M., *Analytica Chimica Acta* (in press).
60. Decristoforo, G. and Knauseder, F., *Analytica Chimica Acta*, **163** (1984), 73.
61. Hayashi, Y., Zaitsu, K. and Ohkura, Y., *Analytica Chimica Acta*, **186** (1986), 131.
62. Koerner, C. A. and Nieman, T. A., *Analytical Chemistry*, **58** (1986), 116.
63. Lundback, H. and Olsson, B., *Analytical Letters*, **18** (B7) (1985), 871.
64. Masoom, M. and Townshend, A., *Analytica Chimica Acta*, **185** (1986), 49.
65. Petersson, B. A., Hansen, E. H. and Ruzicka, J., *Analytical Letters*, **19** (1986), 649.
66. Masoom, M. and Townshend, A., *Analytica Chimica Acta*, **166** (1984), 111.
67. Ibid., **171** (1985), 185.
68. Iob, A. and Mottola, H. A., *Analytical Chemistry*, **52** (1980), 2332.
69. Kiba, N., Tomiyasu, T. and Furusawa, M., *Talanta*, **31** (1984), 131.
70. Gosnell, M. C., Snelling, R. E. and Mottola, H. A., *Analytical Chemistry*, **58** (1986), 1585.
71. Emneus, J., Appelquist, R., Marko-Varga, G., Gorton, L. and Johansson, G., *Analytica Chimica Acta*, **180** (1986), 3.
72. Masoom, M. and Townshend, A., *Analytica Chimica Acta*, **179** (1986), 399.
73. Winquist, F., Lundstrom, I. and Danielsson, B., *Analytical Chemistry*, **58** (1986), 145.
74. Masoom, M. and Townshend, A., *Analytica Chimica Acta*, **174** (1985), 293.
75. Taylor, D. and Nieman, T. A., *Analytica Chimica Acta*, **186** (1986), 91.
76. Olsson, B., Stalbou, B. and Johansson, G., *Analytica Chimica Acta*, **179** (1986), 203.
77. Worsfold, P. J. and Nabi, A., *Analytica Chimica Acta*, **179** (1986), 307.
78. Collis, J. S., Wright, J. M. and Grinman, R. F. A., *Medical Laboratory Science*, **42**, 4 (1985), 310.
79. Olsson, B., *Mikrochimica Acta*, **II** (1985), 211.
80. Ruzicka, J. and Hansen, E. H., *Analytica Chimica Acta*, **179** (1986), 1.
81. Attiyat, A. S. and Christian, G. D., *Analyst*, **105** (1980), 154.
82. Uchiyama, S., Tohfuku, Y., Suzuki, S. and Muto, G., *Bunseki Kagaku*, **35**, 2 (1986), 134.
83. Yao, T., Kobayashi, Y. and Sato, M., *Analytica Chimica Acta*, **153** (1983), 337.
84. Ríos, A., Luque de Castro, M. D. and Valcárcel, M., *Analytical Chemistry*, **58** (1986), 663.
85. Ibid., *Analytica Chimica Acta*, **187** (1986), 139.
86. Ruz, J. (private communication).
87. Zoonen, P. V, Kamminga., D. A., Gooijer, C., Velthorstand, N. H. and Frei, R. W., *Analytical Chemistry*, **58** (1986), 1248.
88. Hool, K. and Nieman, T. A., *Analytical Chemistry*, **59** (1987), 869.
89. Yoshimura, K. and Waki, H., *Talanta*, **32** (1985), 345.

### ADVERTIZING IN 'JOURNAL OF AUTOMATIC CHEMISTRY'

The *Journal* sells internationally – 24% of readers are in the UK; 35% in North America; 25% in Western Europe; 2% in Africa; 4% in Asia; 2% in Eastern Europe; 3% in Australasia; 5% in Japan. Many readers are directly responsible for capital purchases and they fall into the following groups: laboratory managers; clinical, industrial and environmental chemists; manufacturers.

Rates are as follows:

Payment to be made within 30 days of invoice.
Agency commission 15%
Publisher's discount 10%

**Frequency discounts:**

| | 1X | 3X | 4X/6X | 8X | 12X |
|---|---|---|---|---|---|
| Full page B/W | £275 | £270 | £262 | £250 | £245 |
| Half page B/W | £155 | £150 | £145 | £138 | £130 |
| Quarter page B/W | £90 | £87 | £84 | £80 | £75 |

Colour and bleed charges to be added.

**Colour rates:**

Spot colour: £110 extra per page
Four colour: £275 extra per page
Bleed: £45 extra per page.

**Correspondence:**

*All confirmation orders, artwork, film, loose inserts and general correspondence should be addressed to:*
*Mary Hutchins, Advertising Manager, Taylor & Francis Ltd, Rankine Road, Basingstoke, Hampshire RG24 0PR, UK.*
*Tel.: 0256 840366; telex: 858540; fax: 0256 479438.*

